# AKRs confer oligodendrocytes resistance to differentiation-stimulated ferroptosis

**DOI:** 10.1016/j.redox.2024.103463

**Published:** 2024-12-09

**Authors:** Valentina Saverio, Emanuele Ferrario, Romina Monzani, Mara Gagliardi, Francesco Favero, Davide Corà, Claudio Santoro, Marco Corazzari

**Affiliations:** a Department of Health Sciences, School of Medicine, and Center for Translational Research on Autoimmune and Allergic Disease (CAAD), University of Piemonte Orientale, Novara, Italy; b Interdisciplinary Research Center of Autoimmune Diseases (IRCAD), University of Piemonte Orientale, Novara, Italy; c Department of Translational Medicine, School of Medicine, and Center for Translational Research on Autoimmune and Allergic Disease (CAAD), University of Piemonte Orientale, Novara, Italy

**Keywords:** Multiple sclerosis, Ferroptosis, Aldo-keto reductase, AKR1C1, miRNA

## Abstract

Ferroptosis is a recently characterized form of cell death that has gained attention for its roles in both pathological and physiological contexts. The existence of multiple anti-ferroptotic pathways in both neoplastic and healthy cells, along with the critical regulation of iron metabolism involved in lipid peroxides (lipid-ROS) production—the primary mediators of this cell death process—underscores the necessity of precisely controlling or preventing accidental/unwanted ferroptosis. Conversely, dysregulated iron metabolism and alterations in the expression or activity of key anti-ferroptotic components are linked to the development and progression of various human diseases, including multiple sclerosis (MS). In MS, the improper activation of ferroptosis has been associated with the progressive loss of myelinating oligodendrocytes (myOLs). Our study demonstrates that the physiological and maturation-dependent increase in iron accumulation within oligodendrocytes acts as a pro-ferroptotic signal, countered by the concurrent expression of AKR1C1. Importantly, MS-related neuroinflammation contributes to the down-regulation of AKR1C1 through miRNA-mediated mechanisms, rendering mature oligodendrocytes more vulnerable to ferroptosis. Together, these findings highlight the role of ferroptosis in MS-associated oligodendrocyte loss and position AKR1C1 as a potential therapeutic target for preserving oligodendrocyte integrity and supporting neuronal function in MS patients.

## 1 Introduction

Ferroptosis is a recently described new form of cell death, in which the production and accumulation of lipid peroxides, also known as lipid-ROS, represent a key step [1]. Although the precise mechanisms responsible for lipid-ROS toxicity is still under investigation and highly debated, it possibly relies on their integration into phospholipids (mainly PE-OOH) and translocation into cell membrane, thus compromising membrane proteins activity and/or membrane physiology/structure [2]. The production of lipid peroxides within cells is a consequence of various factors such as iron metabolism, lipoxygenase activity, and ROS production. Cells control the levels of lipid-ROS through the action of the main anti-ferroptotic factor GPX4, which converts these highly reactive molecules into less harmful alcohols, using GSH as a cofactor [3].

Beyond GPX4, cells evolved other GPX4/GSH-independent anti-ferroptotic mechanisms although sharing the common target lipid peroxides. Indeed, enhanced expression of FSP1, also known as AIFM2, protects cells from ferroptosis execution by reducing lipid-ROS through the reduction of the ubiquinone CoQ10, thus maintaining ubiquinol/ubiquinone redox balance [4]. While, GCH1 upregulation maintains the balance between BH4 (tetrahydrobiopterin) and BH2 (dihydrobiopterin), with GCH1 promoting the conversion of BH2 back to BH4, which helps in reducing lipid hydroperoxides, thereby inhibiting ferroptosis [5]. On the other hand, the ESCRT III system prevents the accumulation of lipid peroxides and inhibits ferroptosis by facilitating the clearance of damaged membranes, through their active excision. Additionally, ESCRT III mediates the formation of intraluminal vesicles within multivesicular bodies, contributing to the degradation of iron-loaded ferritin and the sequestration of iron, further suppressing ferroptosis [6]. Lipid-ROS can also be inactivated by members of the aldo-keto reductases (AKRs) superfamily of enzymes, as demonstrate by the anti-ferroptotic role of AKR1C1-3 activation/expression in human metastatic melanoma [7,8]. Finally, transglutaminase 2 (TG2 or TGM2) has also been correlated to ferroptosis resistance, although the molecular mechanism is still unclear [9].

While researchers are actively exploring the modulation of ferroptosis as a potential antitumor strategy, there is growing evidence suggesting its involvement in a range of physiological conditions beyond cancer. For instance, cytotoxic CD8^+^ T cells can trigger ferroptosis in cancer cells by affecting specific gene expressions, such as INFγ-dependent downregulation of SLC7A11 and upregulation of ACSL4 [10]. Moreover, aging-related changes in iron and glutathione levels have been linked to a potential induction of ferroptosis in nematodes, while age-related markers of ferroptosis have been detected in brain tissues of aging rats and mice [11].

Therefore, in physiological settings, it is crucial to protect cells from accidental or aberrant stimulation/execution of ferroptosis to prevent the development or progression of diseases. Notably, various ferroptosis markers such as enhanced intracellular labile iron pool (LIP), phospholipid peroxides, and lipid degradation products have been found to be elevated in the brain tissues of patients with multiple sclerosis (MS) and in the EAE mouse model of MS [12,13]. Additionally, recent studies have shown a decreased susceptibility to erastin-induced ferroptosis in maturing human oligodendrocyte cells, yet the exact molecular mechanisms behind this phenomenon remain unclear [14].

It is therefore conceivable that MS-associated myelinating oligodendrocytes (myOLs) loss through ferroptosis might contribute to the progression of the disease. Understanding the molecular mechanism rendering myOLs susceptible to ferroptosis execution will define new valuable potential therapeutic targets to treat MS affected patients to slowdown the progression of this highly invalidating neurodegeneration.

Here we show that oligodendrocyte maturation, involving increasing intracellular iron content, actively induces the early stages of ferroptosis, which is efficiently inhibited by maturation-dependent upregulation of AKRs. Importantly, neuroinflammation observed in MS patients and relying on pro-inflammatory cytokine production results in AKRs deregulation, through miRNA upregulation, thus sensitizing mature OLs to ferroptosis execution.

## 2 RESULTS

Due to the difficulties of working with primary OPCs, their intrinsic heterogeneity, variable differentiation trajectories into myOL, and costs, the use of HOG or MO3.13 cell lines may represent an acceptable compromise to study the biology of OLs and their role in neurodegenerative diseases, at the molecular level, although they fail to achieve the terminal differentiation typical of myOLs.

### 2.1 MO3.13 maturation relay on oxidative stress and confers resistance to general ferroptosis execution

It has been previously suggested that cell maturation might represent a mechanism to protect oligodendrocytes from accidental or disease-ignited ferroptosis execution [14]. To test this hypothesis, we used MO3.13 cells as a model, which display the molecular and cellular features of OL precursors [15].

**Fig. 1:**
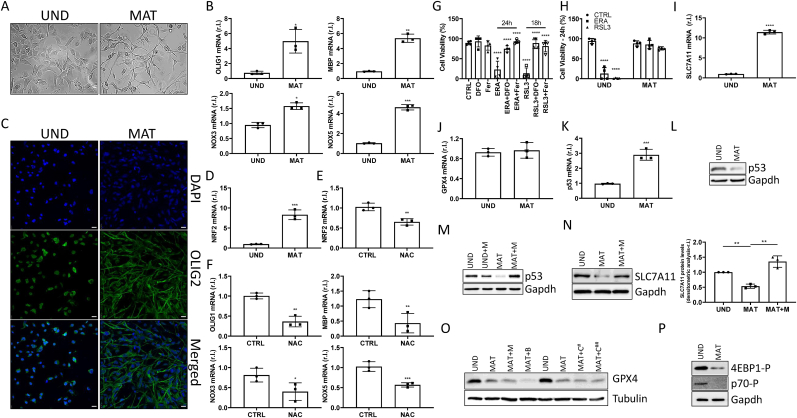
**Oxidative stress-dependent MO3.13 maturation provides resistance against the ferroptotic execution.** (A) Representative images of undifferentiated (UND) and mature (MAT) MO3.13 cells; bar = 100 μm. (B,D) mRNA levels of OLIG1, BMP, NOX3, NOX5 and NRF2 were evaluated by qRT-PCR, in UND and MAT MO3.13. L34 was used as internal control. (C) Representative images of IF staining for oligodendrocyte maturation markers OLIG2 (green), while DAPI was used to stain nuclei, in MO3.13 cells; bar = 25 μm. (E–F) MO3.13 cells were unexposed or exposed to N-acetyl cysteine (NAC; 10 μM) for 3 days, during maturation. mRNA expression levels of NRF2, OLIG1, BMP, NOX3 and NOX5 were evaluated by qPCR. L34 was used as internal control. (G) Undifferentiated cells were exposed to erastin (ERA; 1 μM) or RLS3 (0.5 μM) in presence or absence of Fer-1 (10 μM) or DFO (100 μM), and cell viability was evaluated after 18h. (H) UND and MAT cells were exposed 18h to ERA (1 μM) or RLS3 (0.5 μM), and cell viability was evaluated. mRNA levels of SLC7A11 (I), GPX4 (J) and p53 (K) were evaluated by PCR. L34 was used as internal control. UND and MAT MO3.13 cells were treated or untreated 4h with MG-132 (M; 10 μM) and protein levels of p53 (L) and SLC7A11 (M) were evaluated by western blotting analysis. Tubulin or GAPDH were used as loading control. (N) UND and MAT MO3.13 cells were treated or untreated 4h with MG-132 (M; 10 μM), Bafilomycin (B; 5 nM) or Chloroquine (C; 25 μM and 50 μM) and protein level of GPX4 was evaluated by western blotting analysis. Tubulin was used as loading control. Experiments were performed in triplicate and repeated three times. Histograms represent the mean ± SD; ∗p < 0.05; ∗∗p < 0.01; ∗∗∗p < 0.001; ∗∗∗∗p < 0.0001.

MO3.13 were exposed to PMA and cell maturation was monitored at both morphological and biochemical levels, by measuring the expression of well-known OL markers such as OLIG1, BMP, NOX3, NOX5 and OLIG2. As shown in Fig. 1A–C (and Supplementary S1), the expression of the above-mentioned markers consistently increased in mature MO3.13, paralleled by evident morphological switch.

Oligodendrocyte maturation is associated to increased intracellular ROS accumulation [16], produced by both NOX5 and NOX3, at least in part [15], which results in the upregulation of the antioxidant master transcription factor (TF) NRF2 (Fig. 1D). To confirm the key role played by ROS in the maturation of MO3.13, we observed a decreased expression of NRF2 (Fig. 1E), together with decreased expression of all maturation markers (Fig. 1F), in cells exposed to the antioxidant N-acetyl-cysteine (NAC). Next, to confirm the previously described susceptibility of precursor MO3.13 to ferroptosis execution, we exposed immature cells to the well-known pro-ferroptotic inducers erastin (ERA) and RLS3. As shown in Fig. 1G, cells were extremely sensitive to both compounds, although with a different kinetics. Moreover, to confirm the ferroptotic cell death modality, we used two specific ferroptosis inhibitors such as Deferoxamine (DFO) and Ferrostatin-1 (Fer) [8], which completely inhibited both ERA- or RLS3- induced ferroptosis execution (Fig. 1G). As mentioned above, it has been recently described the resistance of mature MO3.13 to erastin-induced ferroptosis, attributed to maturation-stimulated enhanced expression of SLC7A11 mRNA, the natural target of ERA [14]. We confirmed this feature of mature MO3.13 (Fig. 1H and I), but included RLS3 in our study, to test whether mature cells are specifically resistant to erastin, due to SLC7A11 enhanced expression, or are resistant to ferroptosis in general. Data reported in Fig. 1H clearly show that mature MO3.13 are resistant to both pro-ferroptotic stimuli. To note, RLS3 stimulates ferroptosis by inhibiting GPX4, which is locates downstream to SLC7A11 and which mRNA levels are not altered during cell maturation (Fig. 1J). Therefore, the maturation-stimulated enhanced expression of SLC7A11 mRNA is unable to explain the resistance. However, we decided to go deep in the detail of the molecular mechanism at the basis of the enhanced levels of SLC7A11 mRNA observed during MO3.13 maturation. To this end, we evaluated the potential role of p53, a well-known and well-characterized repressor of SLC7A11 expression, under physiological conditions [17]. We, therefore, compared p53 expression between immature (UND) and mature (MAT) MO3.13, at both mRNA and protein levels. Surprisingly, while p53 mRNA levels increased (Fig. 1K) protein levels decreased dramatically in mature cells, compared to immature ones (Fig. 1L, left panel), due to active protein degradation (Fig. 1L, right panel). In fact, inhibiting proteasomal activity by MG-132 (M), we completely abrogated the maturation-stimulated p53 protein degradation (Fig. 1L, compare MAT with MAT + M lanes). Thus, these data potentially explain the enhanced SLC7A11 mRNA levels in mature MO3.13. However, when we evaluated the protein levels in both immature and mature oligodendrocytes, we observed a clear decrease in SLC7A11, which was mediated by proteasomal degradation (Fig. 1M) [18]. Therefore, these data indicate that SLC7A11 levels are not responsible for mature MO3.13 resistance to ERA-stimulated ferroptosis.

Due to the apparent discrepancy between mRNA and protein levels of both p53 and SLC7A11 in mature MO3.13, we also evaluated the protein levels of GPX4, which mRNA levels were not affected by cell maturation (Fig. 1J), to potentially explain the resistance of mature cells to ferroptosis (Fig. 1H). Surprisingly, GPX4 protein levels were also drastically reduced in mature MO3.13 compared to progenitor cells, which was independent of both proteasomal activity, general autophagy, and CMA (Fig. 1N) [19]. Therefore, collectively these data indicate that neither SLC7A11 nor GPX4 protein levels are responsible for mature MO3.13 resistance to ferroptosis, but a down-stream event must be in place to prevent the execution of this cell death modality.

### 2.2 Maturation-dependent but ferritinophagy-independent iron overload results in cell stress and ferroptosis stimulation

**Fig. 2:**
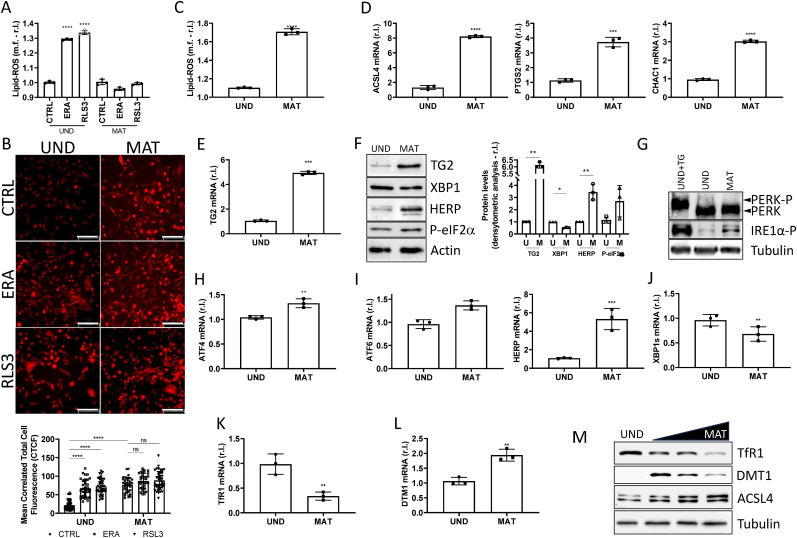
**The maturation-mediated upregulation of ferroptosis markers is independent from ER stress and ferritinophagy.** Immature and mature MO3.13 were exposed 4h to ERA (1 μM) or RLS3 (0.5 μM) and lipid peroxides (A,C) or iron levels (B; in red) were evaluated by BODIPY C11 or FerroOrange probes, respectively. mRNA levels of ACSL4, PTGS2, CHAC1 (D) and TG2 (E) were evaluated by PCR, in UND and MAT MO3.13. L34 was used as internal control. (F) Protein levels of TG2, XBP1, HERP and P-eIF2a were evaluated by western blotting analysis, in the same experimental condition of D-E. Actin was used as loading control. (G) The activation of PERK was evaluated in UND and MAT MO3.13 untreated or treated 2h with thapsigargin (TG), by wb analysis. Tubulin was used as loading control. mRNA levels of ATF4 (H), ATF6, HERP (I), XBP1 (J), TFR1 (K) and DTM1 (L) were evaluated by qPCR, in UND and MAT MO3.13. L34 was used as internal control. (M) UND and MAT MO3.13 cells were treated or untreated 4h with MG-132 (M; 10 μM), Bafilomycin (B; 5 nM), and protein levels of NCOA4 and FTH were evaluated by wb analysis. Tubulin was used as loading control. Experiments were performed in triplicate and repeated three times. Histograms represent the mean ± SD; ∗p < 0.05; ∗∗p < 0.01; ∗∗∗p < 0.001; ∗∗∗∗p < 0.0001.

To determine the molecular mechanism(s) evolved by mature oligodendrocytes to counteract ferroptosis execution, downstream to both SLC7A11 and GPX4, we dissected the signaling pathway. To this aim, we evaluated the generation/accumulation of the key executioners of the ferroptotic process, such as lipid peroxides (or lipid-ROS) [8]. Therefore, proliferating, and mature MO3.13 were exposed to ERA or RLS3 (4h) and lipid-ROS accumulation was evaluated by flow cytometric analysis of BODIPY C11-stained cells. As shown in Fig. 2A, lipid-ROS were increased in sensitive immature MO3.13 cells exposed to ERA/RLS3, while no accumulation was observed in resistant mature cells exposed to neither ERA nor RLS3 (Fig. 2A). Since intracellular iron represents the main source of lipid peroxide, through the Fenton's reactions, we evaluated the labile iron pool (LIP). To this end, both mature and parental cells were stained with the iron specific intracellular probe FerroOrange, and cell's fluorescence was evaluated. Images reported in Fig. 2B clearly show increased intracellular iron in undifferentiated cells exposed to ERA or RLS3, compared to the untreated control (Fig. 2B, upper panels), while no change was observed in mature cells in the same experimental conditions (Fig. 2B, bottom panel). These results are in line with those of Lipid-ROS, reported in Fig. 2A, and indicating no lipid-ROS induction by ERA or RLS3 in mature cells. Moreover, the images in Fig. 2B also show a clear increase in basal LIP in mature MO3.13 (MAT - CTRL) compared to immature cells (UND - CTRL; Fig. 2B, upper and bottom left images), which is not a surprise since mature oligodendrocytes are the cells with the highest iron levels in the brain which is linked to their elevated metabolic needs associated with the process of myelination [20]. Of note, increased LIP in mature MO3.13, compared to immature cells, is also compatible with a pro-ferroptotic status, already indicated by the concomitant decrease in SLC7A11 and GPX4 levels. Therefore, based on the close link between intracellular iron and lipid-ROS, we evaluated the basal level of those molecules in undifferentiated and mature MO3.13. As shown in Fig. 2C, we observed a clear enhanced production of lipid peroxides in mature cells, compared to undifferentiated MO3.13. These results are in line with enhanced expression of typical ferroptotic markers such as ACSL4, PTGS2 and CHAC1 in mature MO3.13 compared to undifferentiated cells (Fig. 2D).

To further verify that increased iron content and consequent lipid-ROS levels represent a stressful condition experienced by mature MO3.13, we evaluated the expression of a typical intracellular stress marker such as TG2 [21], together with the endoplasmic reticulum stress (ER stress), induced by oxidative stress [22], since both of them have been implicated in the ferroptotic signaling pathway [9,23]. Therefore, TG2 mRNA and protein levels were evaluated in both parental and mature MO3.13. Data reported in Fig. 2E and F clearly show enhanced expression of TG2 in mature cells, compared to undifferentiated MO3.13. Next, we verified the induction of ER stress by evaluating the activation of the three arms: the PERK, ATF6 and IRE1α [24]. As shown in Fig. 2F–I, we observed a slight up-regulation of both PERK and ATF6 signaling pathways, as evidenced by: a i) slight phosphorylation of PERK and very low level of activation of the downstream signaling pathway, evidenced by a slight phosphorylation of eIF2α and up-regulation of ATF4; and b) a slight upregulation of both ATF6 and its downstream target HERP. While, analyzing the activation of the IRE1α axes, we observed a down-regulation of its downstream target XBP1, at both mRNA and protein level (Fig. 2F–J).

Collectively, these data are compatible with a chronic ER stress, supporting cell survival, rather than an acute stress and potentially stimulating a cell death process [24].

We also evaluated the potential molecular mechanism responsible for increased iron content of mature oligodendrocytes. To this end, we evaluated the expression of the transferrin receptor TfR1, which is considered the main mechanism for iron uptake and a pro-ferroptotic marker. Our analysis evidenced a decreased expression of TfR1 in mature cells, compared to undifferentiated MO3.13 (Fig. 2K), which is not a surprise since it has been previously reported that TfR is present in OPC and decreases during OL differentiation [20], while no change was observed in the expression of the extracellular iron carrier transferrin (TF; Suppl.S2). It has been previously hypothesized that mOLs might use other factors involved in the iron metabolism to regulate intracellular levels of this element, such as DTM1, FPN or HEPH [20,25]. Our analysis evidenced no change in the mRNA levels of both FPN and HEPH, while increased expression of DTM1 was observed in mature MO3.13 compared to undifferentiated cells (Fig. 2L and Suppl.S2).

Finally, we asked whether the ferritinophagy process, linking iron metabolism and ferroptosis, might contribute to intracellular LIP of mature OLs. Thus, we measured the levels of the ferritinophagy markers NCOA4 and FTH in both undifferentiated and mature MO3.13 and observed a decreased levels of both proteins in mature cells (Fig. 2M), thus indicating a potential induction of the process, while no change was observed at mRNA level (Suppl.S2). However, when we blocked the autophagic process by using Bafilomycin A, no rescue was evident at both NCOA4 and FTH protein levels, while a complete rescue was evident when proteasome activity was inhibited by MG-132 (Fig. 2M). These data indicate that ferritinophagy is not involved in the regulation of intracellular iron levels in mature OLs, while DTM1 might represent the ‘main actor’.

Overall, our data indicate a cellular context (of mature cells) particularly susceptible to ferroptosis and the existence of a potential block of the execution phase, which protects mature oligodendrocytes.

### 2.3 AKR1C1 confers resistance to ferroptosis execution

Although GPX4 represents the main factor regulating the execution of ferroptosis, many cells evolved other mechanisms conferring resistance, independently from GPX4 activity and/or expression, such as the GCH1/BH4 [26], FSP1 [4], and AKRs [8] signaling pathways, which share the common feature of lipid-ROS inactivation.

**Fig. 3:**
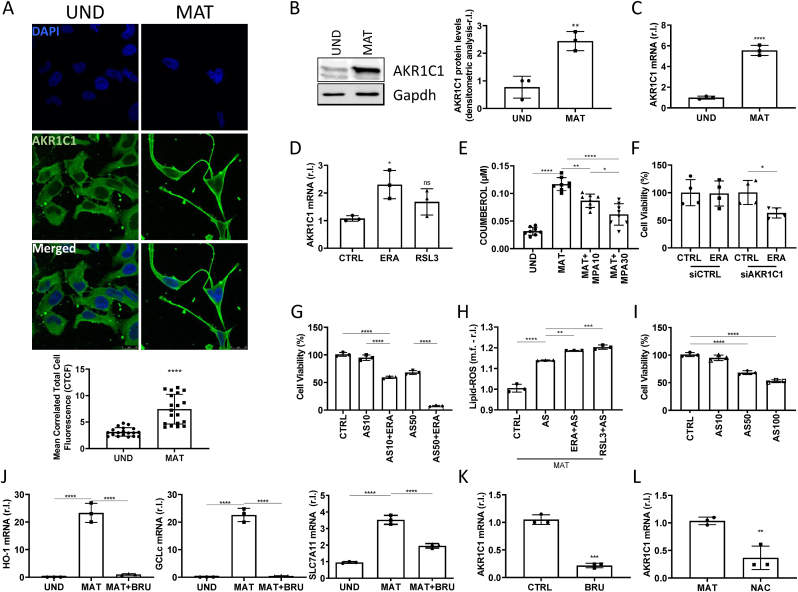
**NRF2-dependent AKR1C1 upregulation confers resistance to ferroptosis execution in mature OLs.** (A) Representative images of immunofluorescence staining for AKRs (green) and DAPI (blue) of undifferentiated (UND) and mature (MAT) MO3.13 cells, while AKRs protein levels were evaluated by wb analysis (B; Tubulin was used as loading control), and mRNA level of AKR1C1 was evaluated by qPCR analysis (C, left panel). Mature cells were unexposed or exposed 8h to erastin (ERA, 1 μM) or RLS3 (0.5 μM), and AKR1C1 mRNA level was evaluated by qPCR (C, right panel). L34 was used as internal control. AKRs enzymatic activity was measured evaluating the conversion of Coumberone into Coumberol in absence or presence of MPA (10 or 30 μM); immature cells were used as negative control. Mature MO3.13 cells were transfected with shRNA targeting AKR1C1 (shAKR1C1), while a scrambled sequence was used as control (shCTRL: E), or were transfected with siRNA targeting AKR1C1 (siAKR1C1), while a scrambled sequence was used as control (siCTRL; F), and cells were exposed to ERA (10 μM) and cell viability was evaluated after 24h. (G) Mature OLs were treated 24h with ERA (1 μM) in combination with 3-bromo-5-phenyl Salicylic Acid (AS; 10 or 50 μM) and the cell viability was evaluated. (H) Mature MO3.13 cells were exposed to ERA (1 μM) or RLS3 (0.5 μM) alone or in combination with AS (10 μM), and Lipid peroxides generation was evaluated after 16 h. (I) Mature OLs were treated 24h with AS (10, 50, 100 μM) and the cell viability was evaluated. (J–K) Mature MO3.13 cells were treated 3 days with or without Brusatol (BRU, 50 mM) and mRNA levels of HO-1, GCLc, SLC7A11 and AKR1C1 were evaluated by qPCR. L34 was used as internal control. (L) MO3.13 cells were exposed or unexposed to NAC, (10 μM) for 3 days, and mRNA levels of AKR1C1 were evaluated by qPCR. L34 was used as internal control. Experiments were performed in triplicate and repeated three times. Histograms represent the mean ± SD; ∗p < 0.05; ∗∗p < 0.01; ∗∗∗p < 0.001; ∗∗∗∗p < 0.0001.

Indeed, we investigated whether one (or more) of those are activated upon maturation of MO3.13. Thus, to evaluate the potential involvement of the GCH1/BH4 system, we measure the mRNA levels of GCH1, which is considered the hallmark of its activation [9], in both undifferentiated and mature MO3.13. Data reported in Supplementary S3 show no change in GCH1 mRNA levels comparing immature and mature cells (Suppl.S3). Similarly, inhibiting the activity of FSP1, by using the specific inhibitor iFSP1, we observed no increased susceptibility of mature MO3.13 to RSL3 (Suppl.S4A). Interestingly, we also observed that FSP1 protein levels decreased upon MO3.13 differentiation (Suppl.S4B), which was time-dependent (Suppl.S4C). Next, we focused on AKR1C1, which is considered the main isoform of the superfamily of AKRs [7] enzymes expressed in the CNS [27]. To this end, we measured the expression of AKR1C1 in undifferentiated and mature MO3.13 at both protein (Fig. 3A and B) and mRNA level (Fig. 3C, left panel). Data reported in Fig. 3A–C shows a clear increased expression of this factor in mature cells. Importantly, both ERA and RLS3 marginally increase AKR1C1 expression of mature cells (Fig. 3C, right panel), thus indicating that maturation represents the primary driver. The maturation-dependent accumulation of AKR1C1 was further confirmed by increased enzymatic activity, compared to immature cells, using MPA (10 μM) as inhibitor [7]. To verify involvement of AKR1C1 in the resistance of mature MO3.13 to ferroptosis execution, we inhibited the expression of the protein by both shRNA and siRNA specific sequences (Suppl.S5A-B, respectively) and evaluated the susceptibility of cells to erastin. As shown in Fig. 3E and F, decreased expression of AKR1C1 sensitized mature MO3.13 to ferroptosis execution. Similar results were obtained by treating mature MO3.13 with erastin in the presence of the AKR1C1 specific inhibitor 3-bromo-5-phenyl Salicylic Acid (AS; Fig. 3G), due to enhanced Lipid-ROS accumulation (Fig. 3H), in a dose-dependent manner. To test whether the maturation-dependent enhanced AKR1C1 expression protects mature oligodendrocytes from increased iron content-stimulated ferroptosis execution, we exposed mature cells to increased concentrations of AS alone, and cell viability was evaluated at 24h post-treatment. Data reported in Fig. 3I clearly show that AKR1C1 activity inhibition ‘*per se*’ consistently reduced the viability of mature cells (Fig. 3I).

Since we previously demonstrated that AKRs expression is under the control of NRF2, in melanoma [7], and that this TF is actively induced during MO3.13 maturation (see Fig. 1), we verified the that NRF2 regulates the expression of AKR1C1 also in mature MO3.13. To this end, mature cells were exposed to the specific NRF2 inhibitor Brusatol (BRU) and the expression of gene targets HO-1, GCLc and SLC7A11 was evaluated by qPCR, using immature cells as a control. Data reported in Fig. 3J show that BRU completely inhibited the NRF2-dependent upregulation of the above-mentioned genes in mature MO3.13, together with the expression of AKR1C1 (Fig. 3K). Similar results were obtained using NAC (Fig. 3L).

We previously demonstrated that the AKR1C1 closely related C2 and C3 members of the AKR superfamily are co-responsible for the resistance of metastatic melanoma to ferroptosis execution [7]. To check if they also have a role in mature oligodendrocytes, we evaluated their expression and activity in MO3.13 cells. Our analysis, reported in Supplementary S6 and S7, show that although the expression of both enzymes increases in a maturation- and NRF2- dependent manner, inhibiting their activity do not re-sensitize mature cells to ferroptosis (Suppl.S6,7).

### 2.4 MS-associated pro-inflammatory cytokines confer sensitivity to ferroptosis through miRNA-dependent down-regulation of AKR1C1

**Fig. 4:**
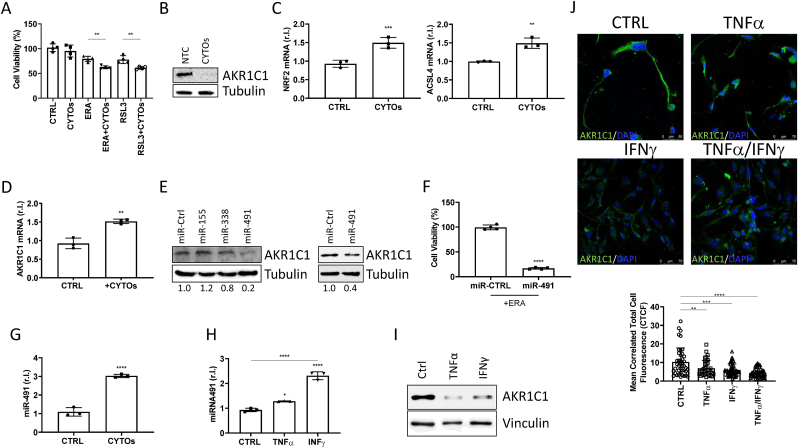
**Pro-inflammatory cytokines sensitize mature OLs to ferroptotic cell death through miRNA-dependent down-regulation of AKR1C1.** (A) Mature OLs were exposed 3 days to pro-inflammatory cytokines (CYTOs; TNF-α 100 ng/ml + IFN-ɤ 100 ng/ml), in the presence or absence of erastin (ERA, 1 μM) or RLS3 (0.5 μM). Cell viability was evaluated after 18 h. Mature MO3.13 were exposed 3 days to cytokines (CYTOs), then (B) AKRs protein expression was evaluated by wb analysis (Tubulin was used as loading control), while mRNA levels of NRF2, ACSL4 (C) and AKR1C1 (D) were evaluated by qPCR. L34 was used as loading control. (E) Huh7 cells were transfected with miRNA-155, miRNA-338 and miRNA-491 or a scrambled sequence (miR-Ctrl), and AKR1C1 protein expression was evaluated by wb analysis. Tubulin was used as loading control. (F) Transfected and mature MO3.13 cells were exposed to ERA (10 μM) and cell viability was evaluated. (G–H) Mature OLs were exposed 3 days to cytokines and the detection of miRNA was performed by qPCR. miRNA103 was used as loading control. (I) Mature OLs were exposed 3 days to cytokines and AKRs protein expression was evaluated by wb analysis. Vinculin was used as loading control. (J) Representative images of immunofluorescence analysis of AKR1C1 (green) and DAPI, in UND and MAT MO3.13 cells exposed 3 days with indicated cytokines. Experiments were performed in triplicate and repeated three times. Histograms represent the mean ± SD; ∗p < 0.05; ∗∗p < 0.01; ∗∗∗p < 0.001; ∗∗∗∗p < 0.0001.

Very recently, the potential involvement of ferroptosis in the pathogenesis of multiple sclerosis (MS) has been reported, although the precise role is still unclear and highly debated [12]. Importantly, the disease is characterized, among others, by the progressive and irreversible loss of mature OLs [28], and we, therefore, asked if ferroptosis might represent the major cause or contribute, using the MO3.13 as a model. To this end, we exposed mature MO3.13 to a mix of the two main and well-characterized pro-inflammatory cytokines IFNγ and TNFα, causing the characteristic MS-associated neuroinflammation, alone or in combination with ERA or RLS3, and cell viability was evaluated at 18h post-treatment. Results reported in Fig. 4A show that the cytokine mix (CYTOs) exacerbates the pro-ferroptotic activity of both ERA and RSL3. Since AKR1C1 expression confers resistance of mature MO3.13 to ferroptosis execution, we evaluated the potential impact of CYTOs on AKR1C1 protein levels. Surprisingly, we observed that CYTOs decreased the expression of this enzyme in mature cells (Fig. 4B), although the expression of NRF2 and its targets genes, such as ACSL4, were upregulated, in the same experimental conditions (Fig. 4C). To note, the latter result is coherent with the known pro-oxidative stress activity of the inflammatory cytokines IFNγ and TNFα [29,30]. However, analysis of AKR1C1 mRNA levels revealed increased expression of this factor, apparently in disagreement with protein levels, observed in the same experimental conditions (Fig. 4D). It is important to note that the CYTOs-mediated reduction in AKR1C1 protein levels is not due to increased protein degradation (Suppl. S10), but rather reflects gene expression regulation influenced by miRNA activity. Indeed, miRNA expression dysregulations have been described in patients affected by MS [31], with miR-155 miR-491 and miR-338 overexpressed in both patients and the EAE mouse model of MS, which also target AKR1C1 [27]. To confirm this last finding, we transiently over-expressed the three miRNAs in the human hepatocellular cell line Huh7, characterized by high levels of AKRs, and observed a reduction in AKR1C1protein levels, particularly in cells expressing miR-491 (Fig. 4E). The latter miRNA was then transiently expressed in mature MO3.13 and cells exposed to erastin. Interestingly, the cell viability assay revealed an enhanced sensitivity of miR-491 expressing mature MO3.13 to ferroptosis, compared to control (Fig. 4F).

Our hypothesis is therefore that MS-associated proinflammatory cytokine production increases the expression of miRNAs targeting AKR1C1, thus sensitizing mature OLs to ferroptosis. To test this hypothesis, we exposed mature MO3.13 to CYTOs and evaluated the expression of miR-491. Data reported in Fig. 4G show an enhanced expression of this miRNA in cells exposed to CYTOs, with the prominent impact of IFNγ (Fig. 4H), which results in enhanced reduction of AKR1C1 protein levels (Fig. 4I and J), compared to TNFα.

### 2.5 Ferroptosis markers and AKRs are increased during OPC-*to*-OL differentiation

As described above, although MO3.13 cells can represent an acceptable surrogate to study the biology of oligodendrocytes at the molecular level, their differentiation *in vitro* is incomplete. Therefore, to validate our results, we analyzed the differentiation trajectory of mouse OPCs into myOLs, by using the online available RNAseq dataset produced by Huan Xu and colleagues [32].

**Fig. 5:**
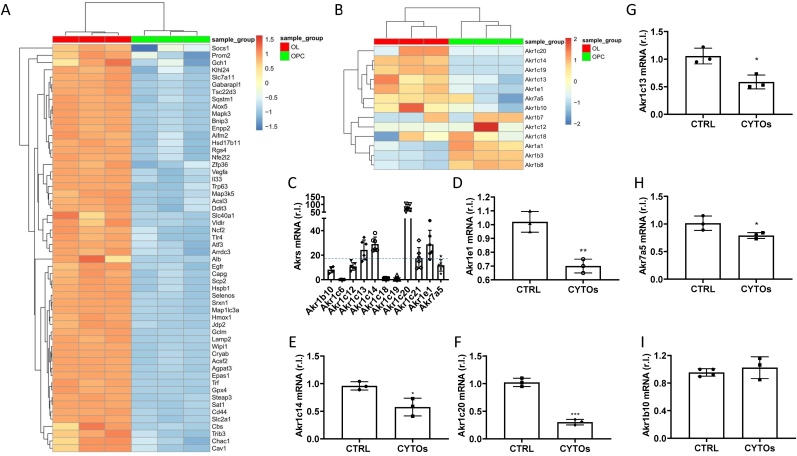
**Ferroptosis markers and AKRs expression increase during murine OPC differentiation.** (A) Heatmap showing Unsupervised Hierarchical Clustering of the 56 upregulated genes in OL vs OPC comparison. (B) Heatmap showing Unsupervised Hierarchical Clustering of the Akr(s) genes expressed in brain tissue. (C) mRNA levels of indicated murine Akr*s* were evaluated by qPCR, in brain tissues of C57BL/6 mice. Gapdh was used as internal control. (D–F) Mice brain tissues were exposed 6 h to cytokines (CYTOs), and the mRNA levels of indicated Akr*s* were evaluated by qPCR. Gapdh was used as loading control. Experiments were performed in triplicate and repeated three times. Histograms represent the mean ± SD; ∗p < 0.05; ∗∗p < 0.01; ∗∗∗p < 0.001; ∗∗∗∗p < 0.0001.

Thus, we evaluated the expression of a list of ferroptosis related genes [33] converting them to the corresponding orthologous mouse ids as described in the materials and methods section. Our analysis revealed the enhanced expression of 56 genes in mouse OLs compared to OPCs, thus confirming a pro-ferroptotic priming associated to oligodendrocyte differentiation (Fig. 5A).

Therefore, to sustain our conclusion that mature OLs are protected against ferroptosis execution through a differentiation-dependent up-regulation of aldo-keto reductases, we evaluated the expression of murine Akr(s) in the above-mentioned dataset. Indeed, although AKR1C1-3 are expressed in human tissues, we screened those expressed in mouse brain and reported in Fig. 5B. Our analysis revealed enhanced expression of Akr1e1, Akr1c13, Akr1c14, Akr1c19 and Akr1c20, which are among those supposed to represent the mouse orthologues of human AKR1C1-3, and the most expressed in the mouse brain [27]. Importantly, we confirmed that at least Akr1e1, Akr1c14, and Akr1c20 are the most expressed in mouse brain, by qPCR (Fig. 5C), which are also downregulated in the presence of CYTOs, *in vitro* (Fig. .5D–F).

## 3 Discussion

The intensive study about the modalities affecting the sensitivity of cancer cells to the execution of the ferroptotic process is revealing the molecular mechanisms evolved by cells to survive in the presence of pro-ferroptotic and therapeutic compounds. And these unvaluable knowledge are changing our perception of ferroptosis. Indeed, accumulating data are unrevealing a ‘physiological’ role of ferroptosis as part of the immune response toward tumor cells and during aging [11]. Moreover, the ubiquitous expression of the main anti-ferroptotic factor GPX4 in human tissues together with the lethality of mouse Gpx4 knockout (Gpx4^−/−^) embryos at E7.5 [34,35], also suggest how important to inhibit accidental or aberrant ferroptosis to sustain physiological cell survival. Therefore, it not a surprise that different cell types activate specific pathways to prevent ferroptotic cell death or that some diseases can be ignited by or may involve the inactivation of one (or more) of those.

A key role in the ferroptotic process is played by iron metabolism, since iron overload stimulates ferroptosis through the production of lipid-ROS [1], the main executioners of this form of cell death. And while most cell types express all the proteins necessary for iron metabolism, in the CNS, however, there is some specialization [36], which raised the hypothesis that each of the CNS cell types has variable needs for iron, and therefore, they distribute and store iron accordingly [37], also indicating that maintenance of iron homeostasis is critical. Indeed, many neurological diseases are characterized by dysregulated iron homeostasis, though in most cases, it is still unclear whether it causes or is a result from these neurodegenerative diseases [37].

In this context, recent studies on MS affected patients and the EAE mouse model of MS, indicated that both iron overload and dysregulated ferroptosis can be involved in the onset and progression of the disease [12,13]. Particularly, ferroptosis might contribute to the MS-associated loss of myOLs, which causes progressive demyelination and neuronal death.

Indeed, intracellular iron increased during oligodendrocytes maturation, in line with increased iron demand necessary for myelin synthesis. Importantly, we found that increased iron represents *per se* a stress stimulus, as evidenced by increased expression of stress-related proteins as TG2 and the induction of pro-survival chronic ER stress [38,39], which induces the early stages of ferroptosis while the execution phase was prevented. Indeed, although immature OLs are sensitive to ferroptosis execution, maturating OLs become resistant, independently from the stimulus. Therefore, an anti-ferroptotic mechanism must be activated during OLs maturation to protect differentiated cells from natural increased intracellular iron and, possibly, from accidental ferroptosis induction.

Screening the anti-ferroptotic pathways potentially conferring resistance to ferroptosis execution of mature OLs we found the key role played by AKR1C1, which expression increases during maturation.

Of note, AKR1C1 plays a key role in regulating neurosteroids in the CNS, metabolites of endogenous steroid hormones that rapidly affect neurotransmitter receptors, modulating neuronal excitability. Indeed, AKR1C1 is involved in the inactivation of 3α,5α-tetrahydro progesterone (3α,5α-THP) and aiding in the synthesis of 3α,5α-tetrahydro deoxycorticosterone (3α,5α-THDOC), known positive allosteric modulators of γ-aminobutyric acid receptors A (GABA_A_) receptors, thereby controlling the levels of GABA receptor modulators in the brain [40].

**Fig. 6:**
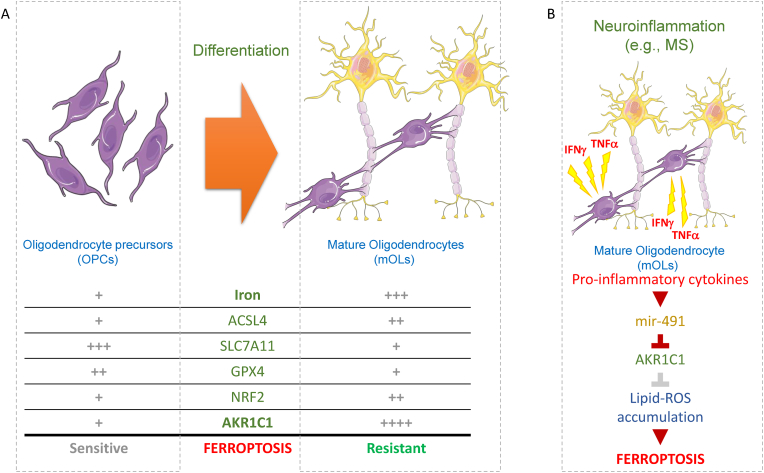
**Ferroptosis and OLs.** A schematic representation of oligodendrocyte differentiation and the gene expression signature that regulates cell sensitivity or resistance to ferroptosis (A). The role of pro-inflammatory cytokines, which are involved in neuroinflammation associated with various neurodegenerative diseases, in modulating mature oligodendrocytes' sensitivity to ferroptosis is also depicted (B). (MS: multiple sclerosis).

Interestingly, AKR inhibition resensitize mature OLs to drug-induced ferroptosis execution. Importantly, ferroptotic cell death was also executed in unstimulated mature OLs, further sustaining the pro-ferroptotic activity of the maturation-dependent increased LIP, and the protective role played by AKR1C1, in healthy OLs (Fig. 6A).

Dysregulated iron metabolism and AKR1C1 activity/expression might, therefore, explain the progressive and irreversible loss of myOLs of patients affected by MS, at least in part. This hypothesis is possibly supported by recent data published by the Christofer Power research group which found a decreased expression of mouse Akr(s) in EAE hindbrain tissues compared with controls [27]. To note, although divergences exist between human and murine homologues of human AKR1C enzymes, akr1c12-14, akr1c18-19 and akr1e1 are the main forms expressed in murine brain tissue [41]. Importantly, the two most abundant isoforms, akr1e1 and akr1c14, showed significant suppression in EAE hindbrains compared with controls [27]. These data were confirmed by our results showing that akr1e1, akr1c14 and akr1c20 were the most abundant isoforms expressed in murine brain and, most importantly, that the expression of the three was consistently reduced in tissues exposed to both IFNγ and TNFα, the main pro-inflammatory cytokines associated with MS-dependent neuroinflammation [42]. Furthermore, downregulated human and murine AKRs was supposed to be caused by dysregulated expression of miRNAs (mainly mir-155, mir-338, and mir-491), evidenced in both MS patients and the EAE mouse model, showing a remarkable degree of similarity between disease-induced micro-RNA changes in human and mouse systems [42]. This hypothesis was supported by our data showing that pro-inflammatory cytokine enhances the expression of (at least) mir-491 which reduces the protein levels of AKR1C1, thus re-sensitizing mature OLs to ferroptosis execution (Fig. 6B).

Finally, analyzing the differentiation trajectory of mouse OPCs into myOLs, a ‘physiological’ induction of the early stages of ferroptosis have been confirmed during cell maturation, paralleled by enhanced expression of protective Akr(s).

Overall, these data unveil the key contribution of ferroptosis in the MS-associated progressive and irreversibly loss of mature OLs, and identify AKRs as new potential therapeutic targets to prevent myOLs integrity and neuronal functionality in patients affected by MS.

## 4 Materials and methods

### 4.1 Cell cultures

The MO3.13 cells (CELLutionBiosystemInc) and Huh7 and HepG2 cell lines were maintained in Dulbecco's Modified Eagles Medium (DMEM; EuroClone), supplemented with 10 % Fetal Bovine Serum (FBS; Merck), 100U/ml penicillin and 100 μg/ml streptomycin (EuroClone), in 5 % CO_2_ at 37 °C.

MO3.13 cells were maturated in FBS-free DMEM, supplemented with 100 nM of Phorbol-12-Myristate-13-Acetate (PMA; Merck), for 3 days.

Cells were treated with erastin 1 μM (ERA; Merck), RSL3 0,5 μM (Merck), Brusatol 50 nM (Bru; Cayman Chemicals), Deferoxamine 100 μM (DFO; Cayman Chemicals), Ferrostatin-1 10 μM (Fer-1; Merck), 3-bromo-5-phenyl salicylic acid 10–50 μM (AS; Merck), Thapsigargin 10 μg/ml (TG; Merck), N-Acetyl-cysteine 10 μM (NAC; Merck), Bafilomycin 10 nM (BAF; Merck); iFSP1 6 μM (BioVision), MG132 10 μM (Merck) and Cloroquine 50 μM (Merck).

Human recombinant TNF-α (100 ng/ml) and IFN-ɤ (100 ng/ml) were purchased from Peprotech.

All agents were added in DMSO, methanol or PBS, with an equal volume of vehicle used to treat control cells.

### 4.2 Mice and treatments

C57BL/6 mice were obtained from Charles River (Calco). Brains were freshly isolated from mice and cultivated in Dulbecco's Modified Eagles Medium (DMEM; EuroClone), supplemented with 10 % Fetal Bovine Serum (FBS; Merck), 100U/ml penicillin and 100 μg/ml streptomycin (EuroClone) and stimulated with TNF-α (100 ng/ml) and IFN-ɤ (100 ng/ml) for 6 h, in 5 % CO2 at 37 °C.

All procedures were approved by the local Ethics Committee for Animal Welfare (IACUC No 178/219-PR) and conformed to the European Community regulations for animal use in research (2010/63 UE).

### 4.3 Quantitative PCR (qPCR)

TripleXtactor reagent (Smobio) was used to isolate total RNA. The AMV Reverse Transcriptase kit (Promega) was used to produce cDNA following the manufacturer's recommendations. Quantitative PCR reactions were performed by using a CFX96 (Bio-Rad) thermocycler. The Maxima SYBR Green qPCR Master Mix (Smobio) was used to produce amplicons during repetitive cycling of the amplification reaction. The melting curve protocol was used to check for probe specificity.

Primer sets for all amplicons were designed using the IDT Primer Quest Tool. The sequences of primers were as follow:

**Table:** 

Gene	Primer sequence (Forward/Reverse)
***human***	
*GPX4*	TGAGGCAAGACCGAAGTA/GAACTGGTTACACGGGAAG
*SLC7A11*	CTGGGTTTCTTGTCCCATATAA/GTTGCCCTTTCCCTCTATTC
*NOX3*	GAGGGTCTCTCCACCATATT/TTGAGGTAGCTCTCGTTAGG
*NOX5*	GGGTGACTCAGCAGTTTAAG/GTGATGGTGCCACTTCTATC
*OLIG1*	CCCAGCAGTAGGATGTAGT/GTCATCCTGCCCTACTCA
*MBP*	CTGTCCCTGAGCAGATTTAG/CCCTTGTGAGCCGATTTAT
*TG2*	CACCCACACCTACAAATACC/CAAAGTCACTGCCCATGT
*ACSL4*	CCTGCAGCCATAGGTAAAG/CAGGCCAGTGTGAAAGAATA
*PTGS2*	GCCTGGTCTGATGATGTATG/GTATTAGCCTGCTTGTCTGG
*CHAC1*	CTCAAGCGCTGTGGATTT/TGTCTCCCTGCCAGAAA
*ATF4*	CCCGGAGAAGGCATCCTC/GTGGCCAAGCACTTCAAACC
*ATF6*	TTTGCTGTCTCAGCCTACTGTGGT/TCCATTCACTGGGCTATTCGCTGA
*XBP1*	AGAGAAAACTCATGGCCTTGTAGTTG/CATTCCCCTTGGCTTCCG
*HERP*	CTCCAGACAGGGATGTACTA/CTGGAAGAAGAGAGGCAAAG
*TfR1*	GTGAGGGATCTGAACCAATAC/TGGAAGTAGCACGGAAGA
*DTM1*	GCTGTCTTCCAAGATGTAGAG/GGATGGGTATGAGAGCAAAG
*FTL*	GAAGCCAGCTGAAGATGA/CAGGGCATGAAGATCCAAA
*FTH*	CTACTGGAACTGCACAAACT/GGCTTTCACCTGCTCATT
*TF*	CTTACCTGGCTCCCAATAAC/ACCACAGCAACAGCATAATA
*FPN*	GGATGGGTCTCCTACTACAA/GCCCAGGACAGTCATATAAAG
*NCOA4*	AGGACCCATGTAAGGTAGAG/GAGCCTCCTTCTCACAATTC
*GCH1*	GTGTATGGTAATGCGAGGTG/GAACTCTTCCCGAGTCTTTG
*AKR1C1*	GCCGTGGAGAAGTGTAAAG/CAGACAGGCTTGTACTTGAG
*AKR1C2*	GGGTTCCACCATATTGATT/CACTGCCATCTGCAATCT
*AKR1C3*	CAGAGGTTCCGAGAAGTAAAG/CCAACCTGCTCCTCATTATT
*NRF2*	TTCCGATGACCAGGACTTA/CAACCCTTGTCACCATCTC
*HO-1*	AGCTCTTCTGGGAAGTAGAC/CCTCCCTGTACCACATCTAT
*GCLc*	CCAAACCATCCTACCCTTTG/TGTTGGCCTCAACTGTATTG
*p53*	TGTACCACCATCCACTACA/TGTTCCGTCCCAGTAGATTA
*L34*	GTCCCGAACCCCTGGTAATAGA/GGCCCTGCTGACATGTTTCTT
***murine***	
*Akr1e1*	TGGATTACCTGGACCTCTAC/CCTCCCAAGTGTCAAGAAAG
*Akr1c6*	CTTCGCTACCAGCTACAAC/GTCCTCTGAAGTCAACTGAA
*Akr1c12*	AATCACTGAAAAGCCTTCAGC/CCCATGTGTCACAGAAATCC
*Akr1c14*	GATACTGGATTCCGCCATTT/CTCTTCACAGTGCCATCTTC
*Akr1c18*	ATGTTGGGTTCTGCCATATT/GTCTGCTCTGGTTGAGATAA
*Akr1c19*	CATGTAGGGCAGGCTATTC/TTTGAGCCCAGGCTTATTC
*Akr1c20*	GCTGGTTTCCGCCATATT/CTCTTCACAGTGCCATCTAC
*Akr1c21*	CGATATGGAGGATGGGTAGA/CAAGGCTGGAGTTCGATTAT
*Gapdh*	TTCAACGGCACAGTCAAG/CCAGTAGACTCCACGACATA

Results were expressed as the threshold cycle (CT). The ΔCT is the difference between the CT for the specific mRNA and the CT for the reference mRNA, L34. To determine relative mRNA levels, 2 was raised to the power of ΔΔCT (the difference between the ΔCT from treated cells and the ΔCT from untreated cells) [43].

### 4.4 miRNA detection

Total RNA was isolated from MO3.13 cells using TripleXtractor reagent (SMOBIO) as described by the manufacturer's protocol. cDNA was synthesized using the miRCURY LNA RT Kit (Qiagen) according to the manufacturer's instructions. 0.5 μL of each spike-in control UniSp6 was added to each reaction to monitor cDNA synthesis efficiency. qPCR was conducted using miRCURY LNA miRNA SYBR Green PCR kit (Qiagen) for miRNA-491, according to the manufacturer's instructions. Quantitative PCR reactions were performed by using a CFX96 (Bio-Rad) thermocycler. The expression of miRNA-491 was normalized to Unisp6spike and miRNA103a-3p. Results were expressed as the threshold cycle (CT). The ΔCT is the difference between the CT for the specific mRNA and the CT for the reference miRNA. To determine relative miRNA levels, 2 was raised to the power of ΔΔCT (the difference between the ΔCT from treated cells and the ΔCT from untreated cells).

### 4.5 Western blotting

Total proteins were extracted by using the RIPA buffer (50 mM Tris-HCl, pH7.5, 150 mM NaCl, 1 % NP40, 0.5 % deoxycholate, 0.1 % SDS) supplemented with a protease inhibitors cocktail (Merck). Cell lysates were resolved by electrophoresis through SDS-PAGE, and electroblotted onto nitrocellulose (Protran, Merck) membranes. Membranes were incubated with indicated primary antibodies in 5 % non-fat dry milk (Bio-Rad) in PBS plus 0.1 % Tween20, overnight at 4 °C.

Primary antibodies were: anti-Olig2 (1:500; Millipore), anti-Tubulin (1:10000; Santa Cruz Biotechnology), anti-p53 (1:500; Santa Cruz Biotechnology), anti-TG2 (1:500; Santa Cruz Biotechnology), anti-PERK (1:500; Cell Signaling Technology), anti-eIF2α-P (1:500; Cell Signaling Technology), anti-XBP1 (1:500; Genetex), anti-HERP (1:2000; Merck), anti-FTH (1:500; Santa Cruz Biotechnology), anti-NCOA4 (1:500; Santa Cruz Biotechnology), anti-FSP1 (1:500; Proteintech), anti-AKRs (1:500; OriGene); anti-SLC7A11 (1:500; Cell Signaling Technology), anti-GPX4 (1:500; Cell Signaling Technology), anti-GAPDH (1:10000; Santa Cruz Biotechnology), and anti-Vinculin (1:10000; Santa Cruz Biotechnology).

Detection was achieved using horseradish peroxidase-conjugate secondary antibody (1:5000; Jackson ImmunoResearch) and visualized with ECL plus (Amersham Biosciences). Images were acquired by using a ChemiDoc™ Touch Imaging System (Bio-Rad) and analyzed by Image Lab software (Bio-Rad).

### 4.6 Immunofluorescence

Cells were grown on glass coverslips, coated with 1 % fibronectin (Merck) and 1 % collagen (Merck), under experimental conditions, as described.

*Olig-2 detection:* cells were fixed in 4 % PFA (Merck) in PBS, supplemented with 2 % Sucrose (Merck), pH 7.4, for 15 min at room temperature. Samples were washes in PBS plus 2 % Sucrose and permeabilized for 10 min at 4 °C with 20 mM Hepes (Merck), 300 mM Sucrose (Merck), 50 mM NaCl (Merck), 3 mM MgCl2 (Merck), plus 0.1 % Triton X-100 (Merck). Cells were then incubated 30 min at 4 °C with blocking buffer consisting in 20 % FBS in PBS. Samples were incubated with primary rabbit polyclonal anti human Olig-2 antibody (Millipore), 1h (rt), washed and labelled with secondary 488 anti-rabbit IgG, 30min, rt.

*AKR staining:* samples were washed with cold PBS and fixed in PFA 4 % for 15 min at 4 °C, permeabilized with 0.5 % Triton X-100 in cold PBS for 10 min at 4 °C, washed twice and incubated with 10 % donkey serum (Jackson ImmunoResearch) plus 0.05 % Triton X-100 in cold PBS, 30 min at 4 °C. Next, the slides were washed and incubated with anti-AKR primary antibody in 1 % donkey serum plus 0.05 % Triton X-100 in cold PBS, 1 h at 4 °C. After washing, the slides were incubated with appropriate secondary antibodies (Jackson ImmunoResearch) 1:500 in 1 % donkey serum plus 0.05 % Triton X-100 in cold PBS and incubated for 1 h at 4 °C.

Coverslips were mounted onto glass using ProLong Gold Antifade with DAPI mounting solution (Thermo Fisher).

Images were acquired using a THUNDER 3D Cell Imager (Leica) or SP8 confocal microscope (Leica).

### 4.7 Lipid peroxides evaluation

Briefly, 35 x 10^3^ cells were treated as indicated and cells harvested at indicate time points. Then, cells were washed with PBS and stained with BODIPY C11 (2 μM in PBS; Invitrogen) 15 min at 37 °C in the dark. Cells were pelleted and resuspended in PBS and 10 x 10^3^ events were acquired by using a FACSymphony (Beckton Dickinson). Data analysis was performed using the Flowing Software.

### 4.8 Cell viability

Cell viability was measured using AlamarBlue™ reagent (Bio-Rad) according to manufacturer's instructions. Briefly, 35 × 10^3^ cells/well were plated in 24-well plates, treated as indicated, cell medium was discarded and an appropriate amount of AlamarBlue reagent was added. Cells were incubated 4 h, and fluorescence was monitored (530–560 nm excitation, and 590 nm emission wavelength) using a TECAN automation platform.

### 4.9 Detection of intracellular Fe^2+^ (LIP)

LIP was measured using the FerroOrange reagent (Dojindo), according to manufacturer's instructions. Briefly, 15 x 10^4^ cells/well were seeded in 6-well plates, treated as indicated, washed twice with PBS and stained with 1 μM FerroOrange in DMEM w/o FBS for 30 min at 37 °C. Then, staining solution was discarded and cells washed with PBS. Images were acquired using a THUNDER 3D Cell Imager (Leica).

### 4.10 **Transfection** (shRNA and miRNA)

15 × 10^4^ cells/well were transfected with 1 μg of DNA in 6-well plates by using JetPRIME (Polyplus) for 8 h, as recommended by the supplier. After 24 h cells were trypsinized, plated and treated as indicated.

To downregulate the expression of AKRs, pLKO-shAKR1C1, pLKO-shAKR1C2, and pLKO-shAKR1C3 were used, while pLKO-shSCRAMBLE was used as a control (Merck).

To ectopically express miRNA, miR-155 (217HmiR0358-MR04-10), miR-338 (217HmiR0290-MR04-10), and miR-491 (217HmiR0137-MR04-10) were used, while a miRNA scrambled control (217CmiR0001-MR04-10) was used as a control (Tebu-Bio).

### 4.11 Small interfering RNA (siRNA)

siAKR1C1, siAKR1C2, siAKR1C3, and non-targeting scramble (siCTRL, used as negative control) siRNA oligoribonucleotides were obtained from Merck. Briefly, 15 × 10^4^ cells/well were seeded in 6-well plate and transfected with 25 pmol of each siRNA, using Lipofectamine®RNAiMAX (Thermo Fisher) reagent, as recommended by the supplier. Cell culture medium was replaced with fresh complete medium after 24 h, cells were lysed after further 24 h, and gene expression analysis was performed by qPCR, as described above.

### 4.12 Measurement of AKRs enzymatic activity

35 x 103 cells/well were seeded in 12-wellplates, and supplemented in complete media (DMEM w/o phenol red plus 100 U/ml penicillin, 100 μg/ml streptomycin, 4 mM l-glutamine, and 10 % FBS) containing Coumberone (10 μΜ; MedChemExpress) ± MPA (10 or 30 μΜ; Cayman Chemicals). The fluorescence of the cell medium was recorded after 3h, by using a 96-wellplate, by a Cytation 5 plate reader (Biotek). Metabolic conversion rate was obtained by detecting fluorescence at 510 nm upon excitation at 385 nm. Coumberol was quantified using 0.1–10 μM coumberol calibration curves in culture medium [44].

### 4.13 RNAseq analysis

We proceeded downloading the gene counts file from the dataset GSE124244 at NCBI GEO portal [32]. We only selected control samples (of both OPC and OL) and we filtered away the genes that have a count equal to “0” in every sample. Ensembl Biomart tool was used to derive the corresponding gene names associated to ENSMUSG ids. Then, DESeq2 R package was used to evaluate a log2FC and an FDR for every gene in the OL vs OPC comparison, in triplicate, selecting log2FC > 1 and FDR <0.05 as thresholds for differentially expressed genes.

To infer mouse orthologous from the list of human ferroptosis related genes [33], we mined the Ensembl biomart database for human/mouse orthologous gene pairs, selecting only genes flagged as “ortholog_one2one” with high confidence level. Heatmaps were produced using the “phetmap” package in R.

### 4.14 Statistical analysis

Experiments were performed in triplicate and repeated at least three times with representative data shown, and statistical analysis was performed using GraphPad software (GraphPad Software; GraphPad Prism 6). Student's *t*-test or ANOVA was used to determine statistical significance. A *p* value of equal to or less than 0.05 was considered significant. mRNA expression levels were represented as ‘fold change’, r.l. relative levels. Histograms represent mean ± SD; ∗∗∗∗p < 0.0001; ∗∗∗p < 0.001; ∗∗p < 0.01; ∗p < 0.05; ns non-significant.

## CRediT authorship contribution statement

**Valentina Saverio:** Writing – original draft, Investigation, Formal analysis, Data curation. **Emanuele Ferrario:** Investigation. **Romina Monzani:** Investigation. **Mara Gagliardi:** Investigation. **Francesco Favero:** Methodology, Formal analysis. **Davide Corà:** Writing – review & editing, Methodology, Formal analysis. **Claudio Santoro:** Writing – review & editing. **Marco Corazzari:** Writing – review & editing, Writing – original draft, Supervision, Project administration, Funding acquisition, Formal analysis, Conceptualization.

## Funding

This work was supported by the Italian Ministry of Education, University and Research (MUR) program “Departments of Excellence 2018–2022”, FOHN Project—Department of Health Sciences, Università del Piemonte Orientale, and MUR Progetti di Ricerca di Rilevante Interesse Nazionale (PRIN) Bando 2022 – grant 202285XS52. The support of FAR 2019 (Progetti di Ateneo), the EU grant “PREMUROSA” (ID #860462), “ExcellMater” (ID #952033) H2020 projects are also acknowledged.

## Declaration of competing interest

The authors declare that they have no known competing financial interests or personal relationships that could have appeared to influence the work reported in this paper.

## Data Availability

All data are reported in the article and supplementary materials.
