# Supplementary material for: AKRs confer oligodendrocytes resistance to differentiation-stimulated ferroptosis
Source: Redox Biol. 2024 Dec 9;79:103463. doi: 10.1016/j.redox.2024.103463 (PMC11699626; doi:10.1016/j.redox.2024.103463)
Supplement: Multimedia component 1 [file mmc1.pdf]

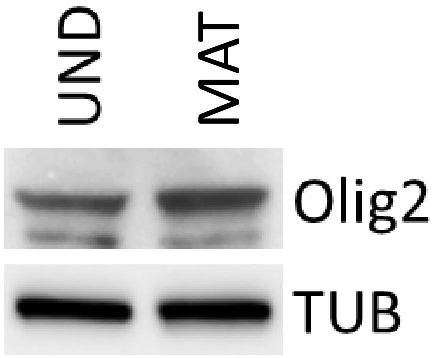


**Supplementary S1. MO3.13 maturation.** MO3.13 cells were maturated as described in the matherial and methods section and the expression of the maturation marker OLIG2 was evaluated by wb analysis. Tubulin was used as loading control. The images are representative of experiments performed three times.

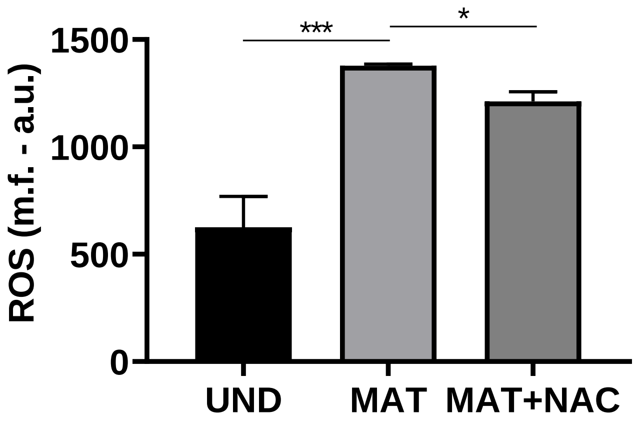


**Supplementary S2. ROS production during MO3.13 maturation.** MO3.13 cells were maturated as described in the matherial and methods section and the production of ROS was evaluated by flow cytometric analysis of H2DCFDA-stained cells. NAC was used as ROS scavanger and cells were exposed to 10µM NAC for 3 days, during maturation.

Histograms represent mean ± s.d of experiments performed in triplicate and repeated al least three times. *** $p < 0.001$; * $p < 0.05$.

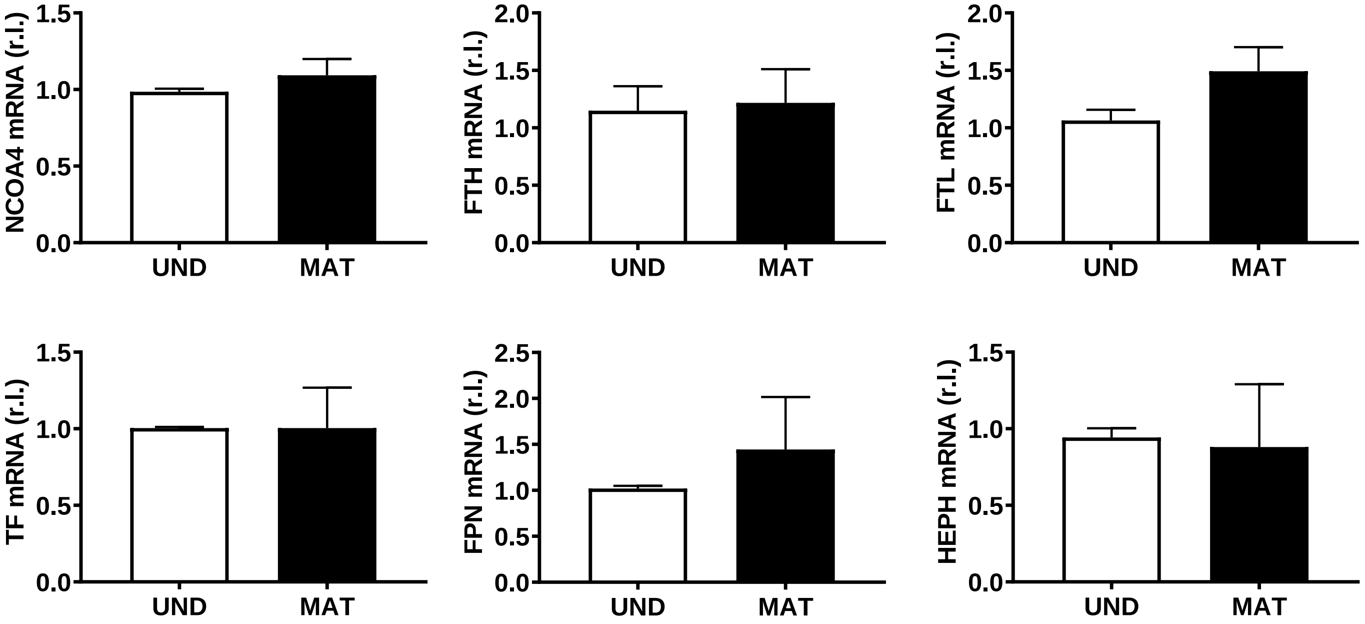


**Supplementary S3. Iron metabolism.** The expression of indicated genes involved in iron metabolism was evaluated in undifferentiated or maturating OLs by qPCR. L34 was used as loading control. Histograms represent mean ± s.d of experiments performed in triplicate and repeated al least three times.

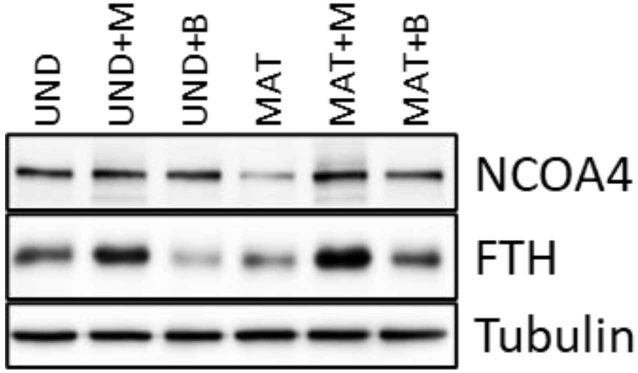


**Supplementary S4. Ferritinophagy analysis.** UND and MAT MO3.13 cells were treated or untreated 4h with MG-132 (M; 10μM), Bafilomycin (B; 10nM), and protein levels of NCOA4 and FTH were evaluated by wb analysis. Tubulin was used as loading control. Experiments were performed in triplicate and repeated three times.

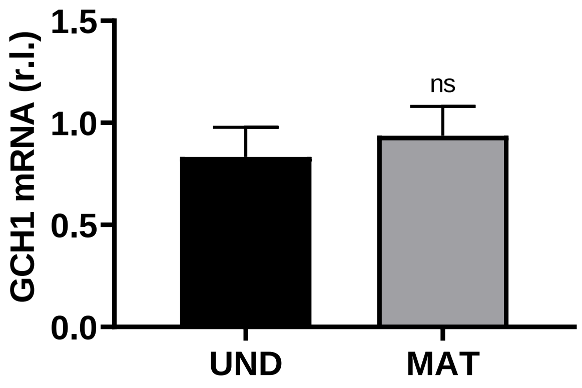


**Supplementary S5. GCH1/BH4.** The expression of GCH1 was evaluated in undifferentiated or maturating OLs by qPCR. L34 was used as loading control. Histograms represent mean ± s.d of experiments performed in triplicate and repeated al least three times. ns = not statistically significant.

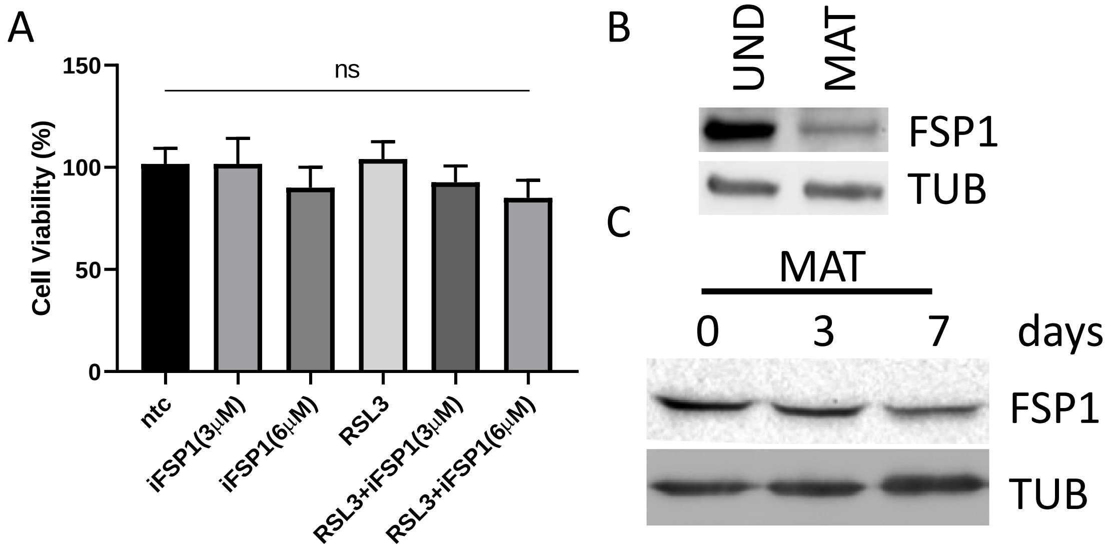


**Supplementary S6. FSP1 analysis. A)** Maturating MO3.13 were exposed to the specific FSP1 inhibitor (iFSP1) at 3 or 6μM or RSL3 (1μM) alone or in combination and cell viability was evaluated at 24h, by AlamarBlue staining. **B)** FSP1 expression was evaluated in immature (UND) or mature (MAT) OLs by WB analysis. **C)** FSP1 expression was evaluated in undifferentiated or maturating OLs for 3 or 7 days. Tubulin was used as loading control. Images are representative of experiments performed three times. Histograms represent mean ± s.d of experiments performed in triplicate and repeated al least three times. ns = not statistically significant.

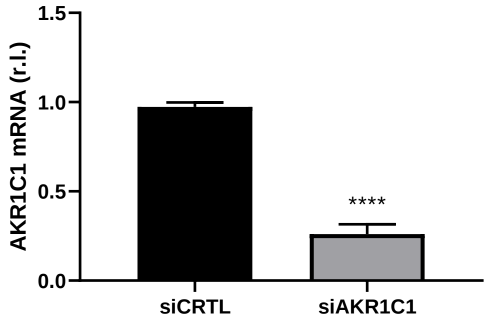


**Supplementary S7.** AKR1C1 silincing. Unifferentiated MO3.13 were transiently transfected with specific siAKR1C1 or scrambled sequences oligos (siCTRL) and AKR1C1 expression was evaluated by qPCR after 3 days of differentiation. L34 was used as loading control. Histograms represent mean ± s.d. of experiments performed in triplicate and repeated al least three times. **** p < 0.0001.

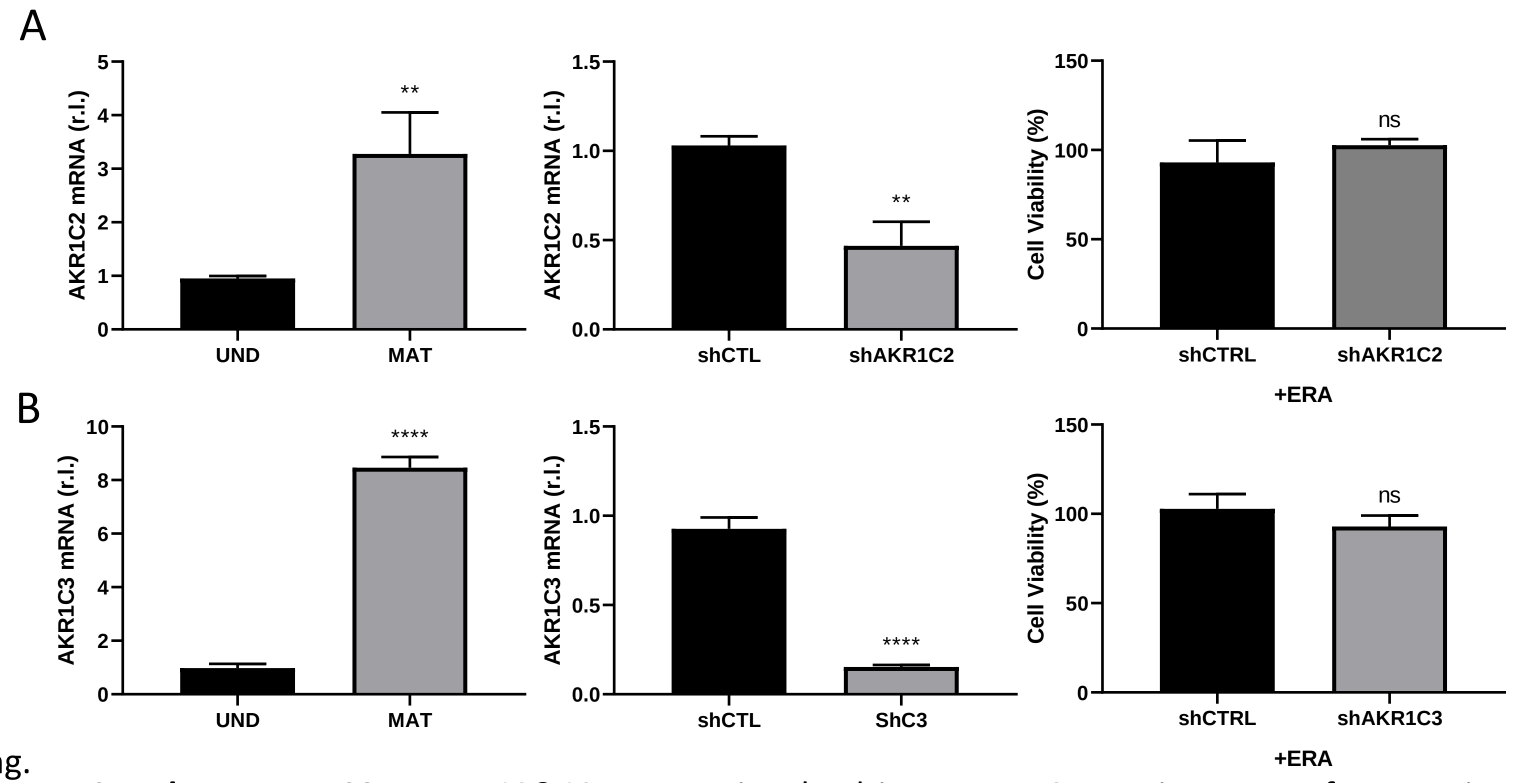


**Supplementary S8.** AKR1 C2&C3 are not involved in mature OLs resistance to ferroptosis execution. AKR1C2 (**A** left panel) or AKR1C3 (**B** left panel) expression was evaluated in both undifferentiated and mature OLs by qPCR. L34 was used as loading control. Next, immature MO3.13 were transiently transfected with vectors coding for specific shAKR1C2, shAKR1C3 or scrambled (shCTRL) sequences and AKR1C2 or AKR1C3 expression was evaluated by qPCR after 3 days of maturation (**A** middle panel, **B** middle panel, respectively). Cells in which the expression of C2 or C3 were downregulated were than exposed or unexposed to ERA (1µM) and cell viability was evaluated at 24h, by AlamarBlue staining (**A** right panel, **B** right panel, respectively). Histograms represent mean ± s.d of experiments performed in triplicate and repeated al least three times. **** p < 0.0001; ** p < 0.01; ns = non statistically significant.

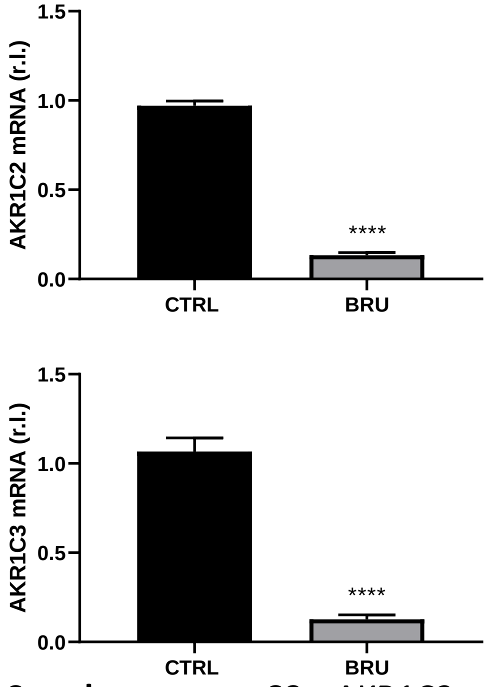


**Supplementary S9.** AKR1C2 and AKR1C3 are transcriptionally regulated by NRF2. Maturating MO3.13 were exposed to Brusatol (BRU, 50mM)) and AKR1C2 (upper panel) or AKR1C3 (bottom panel) expression was evaluated by qPCR. L34 was used as loading control. Histograms represent mean ± s.d of experiments performed in triplicate and repeated al least three times. **** p < 0.0001.

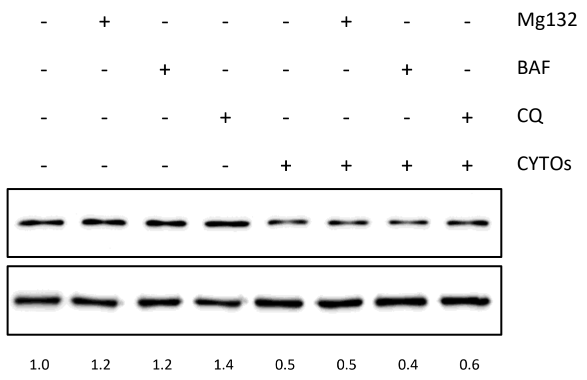


**Supplementary S10.** Mature MO3.13 were exposed to MG132 (10 µM), Bafilomycin (BAF; 10 nM) or Cloroquine (CQ; 50 µM) alone or in combination with pro-inflammatory cytokines (CYTOs; TNF-α 100 ng/ml + IFN-ɤ 100 ng/ml) and the protein levels of AKR1C1 was evaluated (4h) by western blotting analysis. Tubulin was used as loading control. Desitometric analysis was performed and data reported as fold over control.
